# Serum Vitamin D, A, and E Concentrations and Their Associations with Chronic Diseases in Adult Patients Referred to Italian General Practitioners’ Offices

**DOI:** 10.3390/nu18060943

**Published:** 2026-03-17

**Authors:** Paolo Baron, Diego Bigotto, Elena Brignolo, Gabriella Maria Camusso, Alberto Cannavino, Norberto Carli, Francesco Castronuovo, Carmine Colleluori, Provvidenza Fazio, Roberto Ferroni Nichelino, Giorgio Fiorello Chieri, Francesco Fontana, Lino Gambardelli, Patrizia Mascarello, Gabriella Musica, Claudio Nardo, Immacolata Piccirillo, Herbert Rainer, Alberto Rolfo, Stefano Vignando, Sara Cmet, Annarosa Cussigh, Edmondo Falleti, Pierluigi Toniutto

**Affiliations:** 1 Health District of Udine, Azienda Sanitaria Universitaria Integrata Friuli Centrale, 33100 Udine, Italy; baronp54@gmail.com (P.B.); diego.bigotto@gmail.com (D.B.); colleluori@alice.it (C.C.); vignando@1live.it (S.V.); 2 Azienda Sanitaria Locale (ASL) TO5, 10023 Torino, Italy; elena.brignolo@gmail.com (E.B.); mariagrabriella.camusso@gmail.com (G.M.C.); francesco.castronuovo@gmail.com (F.C.); medicinaduegiugno@gmail.com (P.F.); centromediconichelino@gmail.com (R.F.N.); francescofontana57@hotmail.com (F.F.); studiomedigruppo@gmail.com (P.M.); medicinageneraleto5@gmail.com (G.M.); studiomedicorolfo@gmail.com (A.R.); 3 Azienda Sanitaria Locale (ASL) Roma Lazio, 00193 Roma, Italy; alberto.cannavino@gmail.com; 4 Azienda Sanitaria dell’Alto Adige, 39100 Bolzano, Italy; norcarli@gmail.com; 5 Azienda USL di Reggio Emilia, 42122 Reggio Emilia, Italy; lino.gambardelli@alice.it; 6 Azienda Sanitaria Universitaria Giuliano Isontina, 34148 Trieste, Italy; nardoch@gmail.com (C.N.); annarosa.cussigh@asugi.sanita.fvg.it (A.C.); 7 Azienda Sanitaria Locale Caserta, 81100 Caserta, Italy; immapiccirillo@virgilio.it; 8 Azienda Socio-Sanitaria Territoriale dei Sette Laghi, 21049 Tradate, Italy; rainer.herbert@gmail.com; 9 Clinical Pathology, Azienda Sanitaria Universitaria Integrata, University of Udine, 33100 Udine, Italy; sara.cmet@asufc.sanita.fvg.it; 10 Hepatology and Liver Transplantation Unit, Azienda Sanitaria Universitaria Integrata, Department of Medicine, University of Udine, P.zale S.M. della Misericordia 1, 33100 Udine, Italy; edmondofalleti@yahoo.com

**Keywords:** vitamin D, vitamin A, vitamin E, chronic disease, general practitioner

## Abstract

Background: Vitamin deficiencies remain prevalent worldwide and contribute to chronic disease burden. This study evaluated serum concentrations of vitamins D, A, and E in Italian general practice populations and examined their associations with prevalent chronic diseases. Methods: This multicenter cross-sectional study enrolled 500 adult patients (median age: 60.7 years; 40.4% male) attending 21 general practice offices across Italy between January 2021 and December 2024. Serum concentrations of 25-hydroxyvitamin D [25(OH)D], vitamin A, and vitamin E were measured alongside demographic, clinical, and biochemical parameters. Statistical analyses included chi-square tests, correlation analyses, and stepwise logistic regression. Results: The median 25(OH)D concentration was 20.4 ng/mL, below optimal levels. Vitamin D deficiency was significantly associated with osteoporosis (*p* = 0.015), arterial hypertension (*p* = 0.047), and coronary artery disease (*p* = 0.002). The median vitamin A (654 μg/L) and vitamin E (11.3 mg/L) concentrations were within normal ranges. Notably, elevated vitamin A levels were significantly associated with arterial hypertension (*p* = 0.005), diabetes mellitus (*p* = 0.036), and cancer (*p* = 0.010). In the multivariate analysis, elevated vitamin A levels emerged as an independent predictor of multiple comorbidities (*p* < 0.001) and cancer presence (*p* = 0.014), alongside age, male gender, and body mass index. Conclusions: Vitamin D insufficiency is highly prevalent in Italian primary care populations. Vitamin A concentrations show independent associations with multimorbidity and cancer, suggesting a potential negative influence of an animal-based diet, warranting prospective investigation. These findings highlight the importance of comprehensive vitamin assessment in general practice settings.

## 1. Introduction

Vitamins are essential micronutrients that play critical roles in maintaining human health and preventing chronic diseases. Among fat-soluble vitamins, vitamins D, A, and E have been extensively studied for their diverse biological functions, including immunomodulation, antioxidant activity, and cellular differentiation [1,2,3]. Despite their well-established importance, vitamin deficiencies remain prevalent worldwide, contributing to increased morbidity and mortality from chronic non-communicable diseases [4,5].

Vitamin D, primarily recognized for its role in calcium homeostasis and bone metabolism, has emerged as a crucial regulator of multiple physiological processes beyond skeletal health [6,7]. Observational studies have consistently demonstrated an inverse association between vitamin D status and the risk of major chronic diseases, including type 2 diabetes, cardiovascular disease, and cancer [8,9,10]. Recent evidence from large-scale epidemiological studies indicates that vitamin D deficiency affects an estimated 50% or more of populations in both developed and developing countries, with a particularly high prevalence among older adults and specific ethnic groups [2,11]. The 2024 Endocrine Society Clinical Practice Guidelines have updated recommendations for vitamin D assessment and supplementation, emphasizing the need for targeted interventions in high-risk populations while recognizing ongoing debates regarding optimal serum levels and universal supplementation strategies [12,13].

Vitamin A, encompassing both preformed retinol and provitamin A carotenoids, is essential for vision, immune function, cellular differentiation, and epithelial integrity [14,15]. While severe vitamin A deficiency has been largely eradicated in developed countries through food fortification programs, subclinical deficiency and, conversely, excessive intake remain areas of clinical concern [16]. The relationship between vitamin A status and chronic disease exhibits complexity, with both deficiency and excess potentially associated with adverse health outcomes [17,18]. Recent systematic reviews and meta-analyses have revealed mixed findings regarding the association between vitamin A intake or serum levels and cancer risk, with some studies suggesting protective effects for certain malignancies, while others indicate potential harm, particularly in specific populations such as smokers receiving high-dose beta-carotene supplementation [19,20,21]. Furthermore, emerging evidence suggests that elevated serum vitamin A levels may be associated with metabolic dysfunction and chronic inflammatory states, although the directionality of this relationship—whether causative or consequential—remains unclear [22,23,24].

Vitamin E, comprising eight naturally occurring isomers (four tocopherols and four tocotrienols), functions primarily as a lipophilic antioxidant protecting cellular membranes from oxidative damage [25]. Alpha-tocopherol, the most biologically active form, has been studied extensively for its potential role in preventing cardiovascular disease, cancer, and neurodegenerative disorders [26,27]. However, despite compelling biological mechanisms and promising observational data, large randomized controlled trials have generally failed to demonstrate consistent clinical benefits of vitamin E supplementation for chronic disease prevention [28,29]. Recent meta-analyses examining vitamin E anti-inflammatory properties have shown modest reductions in inflammatory biomarkers such as C-reactive protein and interleukin-6, particularly at higher supplementation doses, yet translation of these effects into clinically meaningful outcomes remains elusive [30]. The European Food Safety Authority’s 2024 scientific opinion on tolerable upper intake levels for vitamin E has reinforced concerns about potential adverse effects, particularly increased bleeding risk, emphasizing the importance of understanding both deficiency and excess states in clinical populations [31].

The interplay between vitamin status and chronic disease development is further complicated by multiple factors, including genetic polymorphisms affecting vitamin metabolism, concurrent medications, dietary patterns, and comorbid conditions that alter absorption and utilization [32,33]. Moreover, the metabolic relationships among different vitamins and their roles in shared pathophysiological pathways suggest that assessment of multiple vitamin status simultaneously may provide more comprehensive insights into disease risk than evaluation of individual vitamins in isolation [34].

General practice settings represent an ideal environment for evaluating vitamin status in community-dwelling adults, as general practitioners serve as first-line healthcare providers with access to diverse patient populations across a wide age spectrum. However, limited data exist regarding the distribution of vitamin D, A, and E concentrations in primary care populations and their associations with common chronic comorbidities encountered in clinical practice.

Therefore, this cross-sectional cohort study aimed to evaluate serum concentrations of vitamins D, A, and E in a cohort of adult patients attending general practice offices throughout Italy, and to examine the associations between these vitamin levels and prevalent chronic diseases, including cardiovascular disease, diabetes mellitus, osteoporosis, and malignancy. By characterizing vitamin status and its clinical correlates in this population, we sought to identify patterns that might inform screening strategies and preventive interventions in primary care settings.

## 2. Materials and Methods

### 2.1. Study Protocol

This multicenter cross-sectional cohort study was conducted in collaboration with general practitioners (GPs) across Italy. The study protocol was proposed to multiple GPs working in Northern, Central, and Southern Italy through the Italian GP Federation. Following protocol review, 21 Italian GPs (18 from Northern Italy, 2 from Central Italy, and 1 from Southern Italy) agreed to participate in this study. The protocol was approved by the Internal Review Board of the University of Udine and conducted in accordance with the Declaration of Helsinki. Each enrolled patient provided written informed consent, which was administered by their respective GP. Serum vitamins level measurements and determination of cut-off values of normality were performed in all the laboratories referring to GPs areas following these methods: for 25-OH-vitamin D, by an immunoassay method (<10 ng/mL: deficiency, 10–30: insufficiency, and >30: sufficiency) [2]; for vitamin A, by a reversed-phase HPLC method focused on a retinol-only form (normal values: 300–800 microg/L); and vitamin E, by alpha-tocopherol (normal values: 5.5–18 mg/L) [35].

### 2.2. Study Population

The study population comprised adult subjects aged 18 to 70 years attending the offices of the 18 participating Italian GPs between 1 January 2021 and 31 December 2024. Each GP identified among his patients who did not report taking any vitamin supplements, those in whom at least one measurement of serum vitamins D, A, and E was available during this period. Vitamin measurements may have been performed for specific clinical indications or proposed by the GP as part of a routine health assessment. For each enrolled patient, additional laboratory parameters were collected, including serum levels of parathyroid hormone (iPTH), calcium, phosphorus, uric acid, and creatinine.

After anonymization using numerical codes assigned by each participating GP, the following data were systematically collected from practice management systems: date of data collection, GP identification details, patient demographic characteristics (region of residence, gender, age, and ethnicity, categorized as Caucasian or other), anthropometric measurements (body mass index, calculated as weight in kilograms divided by height in meters squared), occupational status (employed, casual work, unemployed, student, or retired), educational attainment (primary school, middle school, high school, or university (college) degree), smoking history (never, past, or current smoker), and presence of chronic diseases, including malignancy (active or past), cardiovascular disease, stroke, diabetes mellitus, arterial hypertension, respiratory disease, and osteoporosis. All clinical diagnoses and demographic information reflected the status recorded in the patient file at the time of enrollment. Patient age was calculated from the enrollment date.

### 2.3. Data Management

Each participating GP entered the collected data into a standardized Microsoft Excel spreadsheet, with each row representing an individual patient code and each column representing a specific variable. Upon completion of data entry, patient records remained in the possession of the individual GP responsible for patient care. For statistical analysis purposes, only anonymized datasets were transmitted to the study statistician.

### 2.4. Statistical Analysis

Statistical analysis was performed using the Stata 15.1 statistical software (StataCorp, 2017; Stata Statistical Software: Release 15; College Station, TX, USA: StataCorp LLC). Categorical variables were expressed as absolute numbers and percentages, while continuous variables were presented as median values with interquartile ranges. Associations between categorical variables were evaluated using the chi-square test, with the test for linear trend applied when appropriate. The Spearman nonparametric test was used to correlate variables, and the Mann–Whitney test was used to compare the values of vitamins in patients with or without disease. Stepwise logistic regression analysis was utilized to identify independent predictors of vitamins D, A, and E deficiency. A two-tailed *p*-value of less than 0.05 was considered statistically significant.

## 3. Results

Subjects: Five hundred subjects were enrolled in this study. The principal demographic, social, and clinical characteristics of the study population are presented in Table 1. The median age was 60.7 years, and 40.4% of participants were male. Most patients resided in Northern Italy (72.4%), 17.6% were from Central Italy, and 10.0% were from Southern Italy. Approximately 40% were actively employed, while 32.3% were retired. More than half of the enrolled patients had attained higher educational levels, having completed middle school (37.2%) or high school education (30.2%). Nearly one-third of patients had a diagnosis of arterial hypertension, 11.0% had diabetes mellitus, 17.2% had osteoporosis, 7.6% had cancer, 5.4% had coronary artery disease (CAD), and approximately 17.2% were current smokers.

The principal laboratory parameters evaluated at enrollment, including serum concentrations of 25-hydroxyvitamin D [25(OH)D], vitamin A, and vitamin E, are described in Table 2. Regarding vitamin status, only the median 25(OH)D concentration (20.4 ng/mL) fell below the optimal range, whereas median serum concentrations of both vitamin A (654 μg/L) and vitamin E (11.3 mg/L) were within normal reference ranges.

### 3.1. Associations Between Vitamin Concentrations and Clinical Parameters

Table 3 presents the relationships between serum concentrations of vitamins D, A, and E and the principal demographic, clinical, and laboratory parameters.

25-Hydroxyvitamin D: A statistically significant inverse correlation was observed between age and serum 25(OH)D concentrations (*p* < 0.001), which persisted after adjustment for the season in which vitamin D measurement was performed (winter versus summer) (*p* < 0.001). Conversely, the initial significant association between 25(OH)D concentrations and Italian geographic region of residence (*p* < 0.001) was no longer significant following seasonal correction (*p* = 0.238). A significant inverse correlation between 25(OH)D levels and serum PTH was documented (*p* < 0.001), remaining significant after seasonal adjustment (*p* < 0.001). Among social and biochemical parameters, a significant positive correlation was identified between 25(OH)D concentrations and platelet count (*p* = 0.066 before seasonal correction; *p* = 0.004 after seasonal correction).

Vitamin A: Significant positive correlations were found between vitamin A serum concentrations and male gender (*p* < 0.001), body mass index (*p* < 0.001), serum creatinine (*p* < 0.001), serum uric acid (*p* < 0.001), serum calcium (*p* < 0.001), white blood cell count (*p* < 0.001), hemoglobin concentration (*p* < 0.001), and platelet count (*p* < 0.001).

Vitamin E. Significant positive correlations were observed between vitamin E serum concentrations and age (*p* < 0.001), Italian geographic region of residence (*p* < 0.001), serum PTH (*p* = 0.002)—which remained significant after seasonal correction (*p* = 0.009)—as well as hemoglobin concentration (*p* < 0.001) and platelet count (*p* < 0.001).

### 3.2. Vitamin Concentrations and Clinical Diseases

Table 4 presents the correlations between serum concentrations of vitamins D, A, and E and the clinical characteristics of the study population.

Vitamin D: Median serum 25(OH)D concentrations were significantly lower in patients with osteoporosis compared with those without (18.1 vs. 21.1 ng/mL; *p* = 0.015), in patients with arterial hypertension versus those without (19.2 vs. 21.1 ng/mL; *p* = 0.047), and in patients with CAD versus those without (13.7 vs. 20.6 ng/mL; *p* = 0.002).

Vitamin A: Significantly elevated median serum vitamin A concentrations were observed in patients with arterial hypertension compared with those without (670 vs. 647 μg/L; *p* = 0.005), in patients with diabetes mellitus versus those without (693 vs. 650 μg/L; *p* = 0.036), and in patients with cancer versus those without (727 vs. 650 μg/L; *p* = 0.010). Patients with vitamin A over 800 μg/L have a higher cancer presence (15/102 [14.7%] vs. 23/398 [5.8%]; *p* = 0.002).

Vitamin E: The only statistically significant correlation was higher vitamin E serum concentrations in patients presenting osteoporosis compared with those who did not (18.1 vs. 11.0 mg/L; *p* = 0.008).

Table 5 displays the univariate and multivariate analyses examining independent predictors of having no or one (N = 412) or more (N = 88) baseline clinical diseases in the study population. In univariate analysis, age (*p* < 0.001), male gender (*p* < 0.001), living region (*p* = 0.001), education level (*p* < 0.001), job (*p* = 0.003), BMI (*p* < 0.001), creatinine (*p* < 0.001), uric acid (*p* < 0.001), phosphorus (*p* = 0.0127), and vitamin A (*p* = 0.002) were significantly associated with multiple chronic diseases. In multivariate analysis, independent predictors of having multiple clinical diseases included older age (*p* < 0.001), male gender (*p* = 0.003), increased BMI (*p* = 0.021), and elevated serum vitamin A concentrations (*p* < 0.001). A linear trend was detected between the number of patients with a vitamin A serum concentration above the upper limit and the presence of one or more chronic diseases (no disease= 16.3%, one = 19.7%, and two or more = 27.3%; linear trend *p* < 0.001 Supplementary Figure S1).

**Figure 1:**
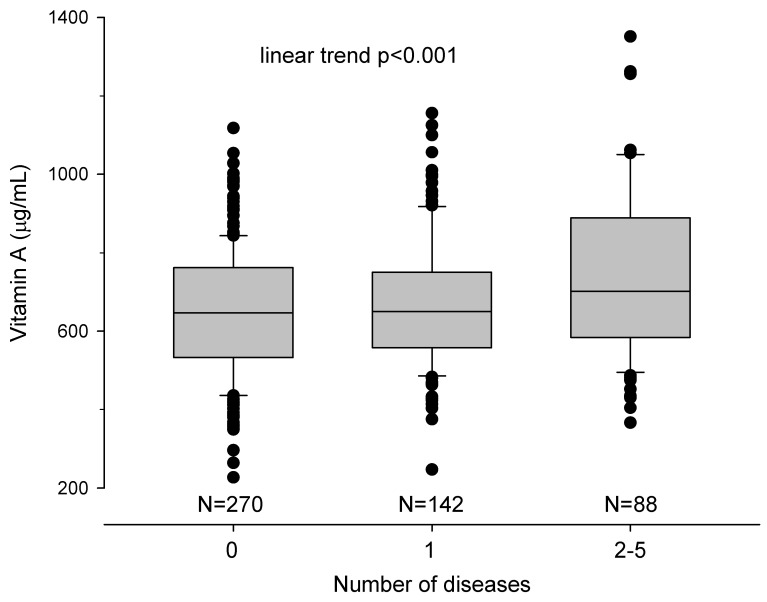
Box plots referring to the median (25th and 75th percentiles) of vitamin A serum levels and the number of diseases present at the baseline in the studied population. Statistical analysis was performed by chi-square for trend.

Furthermore, as shown in Table 6, elevated vitamin A serum concentrations, in addition to older age, emerged as independent predictors of prevalent cancer at baseline in the multivariate analysis. In the univariate analysis, age (*p* < 0.001), male gender (*p* = 0.022), living region (*p* = 0.018), creatinine (*p* = 0.048), uric acid (*p* = 0.023), and vitamin A (*p* = 0.010) were associated with cancer presence. In the multivariate model, only age (*p* < 0.001) and vitamin A (*p* = 0.014) remained as independent predictors. Additionally, a progressive increase in vitamin A serum concentrations was significantly associated with an increasing number of chronic diseases present at baseline (Figure 1), demonstrating a potential dose–response relationship between vitamin A levels and multimorbidity.

## 4. Discussion

This cross-sectional cohort study examined serum concentrations of vitamins D, A, and E in a cohort of 500 adult subjects attending general practice offices throughout Italy, along with their associations with demographic characteristics, clinical comorbidities, and biochemical parameters. Our findings reveal distinct patterns for each vitamin, with important clinical and public health implications.

Regarding 25(OH)D, our results demonstrated that median serum concentrations (20.4 ng/mL) fell below the optimal range, consistent with the widespread vitamin D insufficiency reported in European and global populations [2,11]. The inverse correlation between vitamin D concentrations and age observed in our study aligns with previous literature demonstrating age-related decline in vitamin D status attributable to reduced cutaneous synthesis, decreased renal hydroxylation capacity, and diminished outdoor activity among older adults [6,7]. Importantly, this association persisted after seasonal correction, suggesting that age-related factors beyond sun exposure contribute to vitamin D deficiency. The inverse relationship between 25(OH)D and parathyroid hormone concentrations confirms the well-established negative feedback mechanism in calcium–phosphate homeostasis [13,32].

Our findings of significantly lower vitamin D concentrations in patients with osteoporosis, arterial hypertension, and coronary artery disease are consistent with extensive epidemiological evidence linking vitamin D deficiency to multiple chronic conditions [8,9,10]. This evidence could be reinforced considering that a patient with chronic disease spends more time indoors than a healthy person, thus reducing the self-synthesis of vitamin D during sun exposure [36]. The association with osteoporosis is well documented, as vitamin D plays a crucial role in calcium absorption and bone metabolism [33,34]. Recent meta-analyses have confirmed that combined calcium and vitamin D supplementation modestly improves bone mineral density and may reduce fracture risk in postmenopausal women with osteoporosis, although the magnitude of benefit remains subject to debate [37,38]. The relationship with cardiovascular diseases, including hypertension and coronary artery disease, supports the hypothesis that vitamin D exerts pleiotropic effects beyond skeletal health, potentially through modulation of the renin–angiotensin–aldosterone system, endothelial function, and inflammatory pathways [9,10]. However, the 2024 Endocrine Society guidelines acknowledge that despite compelling observational associations, most randomized controlled trials have failed to demonstrate clear clinical benefits of vitamin D supplementation for cardiovascular disease prevention in the general population [12]. These findings underscore the importance of monitoring vitamin D status in populations at risk for these conditions while recognizing the complexity of translating observational associations into effective interventions.

The vitamin A findings present a more complex and clinically intriguing picture. While median serum concentrations were within the normal range (654 μg/L), we observed significantly elevated concentrations in patients with arterial hypertension, diabetes mellitus, and cancer. The positive correlations between vitamin A concentrations and male gender, BMI, creatinine, uric acid, and hematological parameters suggest that vitamin A metabolism may be influenced by metabolic and inflammatory status [22,23,24]. Most notably, elevated vitamin A emerged as an independent predictor of both multiple comorbidities and cancer presence at baseline, even after adjustment for age, gender, and BMI. This finding requires careful interpretation, as the relationship between vitamin A and chronic diseases exhibits a U-shaped curve, with both deficiency and excess being potentially harmful [17,18]. The association between elevated vitamin A concentrations and cancer deserves particular attention and warrants mechanistic investigation. While vitamin A and its metabolites have traditionally been considered protective against carcinogenesis through their roles in cellular differentiation and immune function [14,15], recent evidence suggests that excessive vitamin A intake or elevated serum retinol concentrations may paradoxically increase cancer risk in certain populations [19,20,21]. Our observation that vitamin A concentrations progressively increased with the number of chronic diseases suggests that elevated vitamin A may serve as a biomarker of underlying metabolic dysfunction or chronic inflammatory states rather than a causative factor. Alternatively, reverse causation cannot be excluded, as chronic diseases may alter vitamin A metabolism, distribution, and clearance. The documented association between vitamin A and metabolic syndrome components, including insulin resistance and adiposity, provides a potential mechanistic link [39,40,41].

The positive correlations between vitamin A and markers such as creatinine, uric acid, and metabolic parameters may reflect hepatic and renal function alterations, as both organs play critical roles in vitamin A metabolism [22]. The liver stores approximately 80–90% of total body vitamin A as retinyl esters, and impaired hepatic or renal function can lead to altered vitamin A homeostasis [23]. Furthermore, insulin resistance and metabolic syndrome, conditions commonly associated with the comorbidities observed in our population, have been linked to altered retinoid metabolism and elevated circulating retinol-binding protein 4 (RBP4), which has emerged as an adipokine implicated in insulin resistance [39,40,41]. A further potential explanation of our findings is to consider that vitamin A per se cannot be the primary factor in determining the increased risk of chronic disease and cancer, but that products present in drinks or foods containing vitamin A may have a relevant role. There are two types of dietary sources of vitamin A: either plant foods containing β-carotene or animal foods containing retinyl esters. It has been shown that the conversion of β-carotene to retinol is under negative-feedback control, since a high vitamin A status inhibits the formation of the rate-limiting enzyme of this conversion [42,43]. This limits the level of retinol in the plasma, even if only part of the dietary vitamin A comes from plant sources. Thus, elevated vitamin A status is only possible if the animal foods in the diet provide an excess of the required retinol. However, the association between high vitamin A serum levels and diseases could be caused either by the vitamin A itself, by anything harmful that occurs primarily in animal-based foods, such as saturated fats or nitrosamines, or by a deficiency of something that does not primarily occur in animal-based food, such as fiber or phytochemicals [44].

The vitamin E results demonstrated a positive correlation with age and PTH concentrations, but, notably, no significant associations were found with clinical comorbidities besides osteoporosis. Vitamin E serum concentrations are regulated by a negative-feedback mechanism, ensuring that the plasma status matches the requirements [45]. Older people, particularly those with osteoporosis, may require more vitamin E to maintain the oxidative balance in their cells than people who do not have these risk factors. In this case, the high vitamin E serum levels would be a marker of oxidative dysfunction, rather than a cause of it [46]. The modest positive correlation between vitamin E and hematological parameters (hemoglobin and platelet counts) may also reflect the antioxidant role of vitamin E in protecting blood cells from oxidative damage [25], although this association lacks clinical significance in the absence of correlations with clinical endpoints. The lack of meaningful associations between vitamin E and chronic diseases contrasts with some earlier observational studies suggesting protective effects of vitamin E against cardiovascular disease and certain cancers [26,27]. However, our findings align with more recent large-scale randomized controlled trials that have failed to demonstrate clear clinical benefits of vitamin E supplementation in chronic disease prevention [28,29]. The ATBC trial and subsequent studies revealed either no benefit or potential harm from high-dose vitamin E supplementation [28].

The multivariate analysis identifying older age, male gender, elevated BMI, and elevated vitamin A concentrations as independent predictors of multiple comorbidities highlights the interconnected nature of metabolic health and vitamin status. The specific association between vitamin A and multimorbidity, independent of other traditional risk factors, suggests that vitamin A metabolism may be more intimately linked to systemic metabolic dysfunction than previously recognized. This finding warrants prospective studies to determine whether elevated vitamin A represents a consequence of chronic disease, a contributor to disease progression, or both. Given the observational nature of our study, we cannot establish causality, and the observed association may reflect confounding by unmeasured factors or reverse causation.

Several limitations of this study merit consideration. First, the cross-sectional design precludes causal inferences regarding the relationships between vitamin concentrations and chronic diseases. Prospective longitudinal studies are needed to establish temporal relationships and determine whether vitamin status predicts disease incidence or represents a consequence of existing disease. Second, the study population was predominantly drawn from Northern Italy, potentially limiting generalizability to other geographic regions or ethnic groups with different dietary patterns, sun exposure, and genetic backgrounds. Third, single-point measurements of vitamin concentrations may not accurately reflect long-term vitamin status, and seasonal variations in vitamin D, despite statistical adjustments, remain a potential source of bias. Finally, residual confounding from unmeasured variables, such as physical activity levels and genetic factors affecting vitamin metabolism, cannot be entirely excluded despite multivariate adjustment.

Despite these limitations, our study has several notable strengths. The relatively large sample size (n = 500) and inclusion of multiple sites across different Italian regions enhance the representativeness of our findings for primary care populations. The simultaneous assessment of three distinct vitamins provides a more comprehensive picture of vitamin status than studies focusing on individual vitamins. Additionally, the inclusion of diverse clinical and laboratory parameters, including hematological indices, renal function markers, and metabolic parameters, allowed for extensive exploration of potential associations and confounding factors.

## 5. Conclusions

In conclusion, this study demonstrates that vitamin D insufficiency is highly prevalent in Italian general practice populations, affecting most surveyed individuals with a median concentration below recommended thresholds. Although vitamin A concentrations were within normal ranges on average, they showed significant and independent associations with multiple chronic diseases and comorbidities, including cancer, diabetes mellitus, and arterial hypertension. The specific relationship between elevated vitamin A and multimorbidity suggests complex metabolic interactions that warrant further investigation through prospective cohort studies and mechanistic research. Vitamin E showed limited associations with clinical outcomes in this population, consistent with recent trial evidence [14] questioning its clinical utility for chronic disease prevention. These findings highlight the importance of comprehensive vitamin status assessment in primary care settings and suggest that vitamin A metabolism may represent an underappreciated marker of metabolic health and disease burden. Future prospective studies with repeated measurements, detailed dietary and supplement data, assessment of genetic variants affecting vitamin metabolism, and mechanistic investigations are needed to clarify the causal relationships between vitamin status and chronic disease development, ultimately informing evidence-based screening and intervention strategies in general practice populations.

In the meantime, it remains of paramount importance to strictly adhere to dietary guidelines, avoiding a delayed implementation of existing intervention strategies, when the current results do not indicate any deficiencies in them.

## Figures and Tables

**Table 1:** Main demographic, social and clinical characteristics of the studied subjects. Categorical variables are reported as frequencies (%), and continuous variables are reported as medians (interquartile ranges).

	N = 500
Age (years)	60.7 (48.4–71.9)
Male gender	202 (40.4)
BMI (Kg/m^2^)	25.0 (22.4–28.0)
Living region	
North Italy	362 (72.4)
Central Italy	88 (17.6)
South Italy	50 (10.0)
Job type	
Stable employment	164 (32.9)
Occasional work	60 (12.0)
Not working	101 (20.2)
Student	13 (2.6)
Retired	161 (32.3)
Education level	
Primary school	109 (21.8)
Middle school	186 (37.2)
High school	151 (30.2)
College	54 (10.8)
Presence of cancer	38 (7.6)
CAD	27 (5.4)
History of stroke	11 (2.2)
Presence of diabetes	55 (11)
Respiratory diseases	28 (5.6)
Arterial hypertension	173 (34.6)
Osteoporosis	86 (17.2)
Active smoking	86 (17.2)

BMI: body mass index; CAD: coronary artery disease.

**Table 2:** Baseline main biochemical parameters and serum vitamin levels of the studied subjects. The variables are reported as medians (interquartile ranges).

	N = 500
Hemoglobin (g/dL)	13.8 (13.0–14.7)
MCV (fL)	91.2 (88.5–94.4)
Platelets (n * 1000/mL)	222 (190–258)
WBCs (n * 1000/mL)	6.01 (4.99–7.26)
Creatinine (mg/dL)	0.88 (0.77–1.00)
Uric acid (mg/dL)	4.6 (3.8–5.6)
Ca (mMol/L)	2.36 (2.30–2.43)
P (mMol/L)	1.07 (0.95–1.19)
iPTH (pg/mL)	57.6 (43.5–78.5)
25-OH-vitamin D (ng/mL)	20.4 (14.0–26.1)
Vitamin A (μg/L)	654 (550–771)
Vitamin E (mg/L)	11.3 (8.2–14.1)

MCV: mean corpuscular volume; WBCs: white blood cells; Ca: serum calcium, P: serum phosphorus; iPTH: serum parathormone.

**Table 3:** Vitamin D, vitamin A, and vitamin E serum concentrations in relation to demographic, clinical, and biochemical parameters in the studied population. Spearman’s rank correlation coefficient rho (r) was calculated and is presented.

	25-OH-Vitamin D		25-OH-Vitamin D (Corrected for Season)		Vitamin A		Vitamin E	
	Corr. Coeff. ρ	*p*	Corr. Coeff. ρ	*p*	Corr. Coeff. ρ	*p*	Corr. Coeff. ρ	*p*
Age (years)	−0.2010	<0.001	−0.1533	<0.001	0.0788	0.0785	0.2785	<0.001
Gender (f = 0/m = 1)	−0.0030	0.9465	0.0065	0.8843	0.2023	<0.001	−0.0015	0.9726
Living region North Italy (0) Central Italy (1) South Italy (2)	0.1950	<0.001	0.0528	0.2383	−0.0888	0.0472	−0.4578	<0.001
Education (0 = primary, 1 = middle, 2 = high, or 3 = university)	0.0776	0.0829	0.0706	0.1148	−0.0185	0.6806	−0.1202	0.0071
Job (0 = no, 1 = occasional, 2 = stable, 3 = retired, or 4= student)	−0.0430	0.3373	0.0007	0.9878	−0.0073	0.8713	0.0733	0.1017
BMI (Kg/m^2^)	−0.0398	0.3743	−0.0330	0.4621	0.2157	<0.001	0.0351	0.4336
Smoking (no = 0/y = 1)	0.0215	0.6309	0.0188	0.6744	0.1243	0.0054	−0.0277	0.5360
Creatinine (mg/dL)	−0.0506	0.2589	−0.0138	0.7584	0.3098	<0.001	0.0662	0.1395
Uric acid (mg/dL)	−0.0334	0.4556	−0.0177	0.6937	0.3186	<0.001	0.0560	0.2115
Ca (mMol/L)	0.0781	0.0812	0.0755	0.0916	0.2133	<0.001	0.0601	0.1796
P (mMol/L)	0.0595	0.1841	0.0164	0.7143	−0.0846	0.0587	0.0281	0.5301
iPTH (pg(mL)	−0.3407	<0.001	−0.3007	<0.001	−0.1030	0.0212	0.1366	0.0022
iPTH (pg(mL) season-corrected)	−0.3303	<0.001	−0.3072	<0.001	−0.1060	0.0178	0.1153	0.0099
WBCs (n * 1000/mL)	−0.0046	0.9174	−0.0122	0.7851	0.2702	<0.001	−0.0379	0.3977
Hemoglobin (g/dL)	0.0209	0.6412	0.0614	0.1705	0.2386	<0.001	0.1480	<0.001
Platelets (n * 1000/mL)	0.084	0.0658	0.1283	0.0041	0.1489	<0.001	0.1793	<0.001

BMI: body mass index; WBCs: white blood cells; Ca: serum calcium, P: serum phosphorus; iPTH: serum parathormone.

**Table 4:** 25-OH-vitamin D, vitamin A and vitamin E serum concentrations in relation to the presence of diseases evaluated in the studied population. Medians (interquartile ranges) and rank-sum test (Mann–Whitney) comparisons are presented.

	25-OH-Vitamin D (ng/mL)			Vitamin A (mg/L)			Vitamin E (mg/L)		
	Present	Absent	*p*	Present	Absent	*p*	Present	Absent	*p*
Osteoporosis (N = 107)	18.1 (11.6–25.6)	21.1 (14.9–26.2)	0.0146	670 (570–800)	645 (544–766)	0.0601	18.1 (11.6–25.6)	11.0 (7.8–13.8)	0.0082
Arterial hypertension (N = 173)	19.2 (12.8–25.1)	21.1 (15.0–26.4)	0.0473	670 (570–808)	647 (532–758)	0.0047	11.8 (9.0–14.3)	10.8 (7.8–14.0)	0.0846
Diabetes (N = 55)	22.3 (13.5–28.3)	20.2 (14.1–26.0)	0.2715	693 (551–905)	650 (548–763)	0.0363	11.2 (7.6–13.7)	11.3 (8.4–14.2)	0.4409
Respiratory diseases (N = 28)	18.8 (12.4–24.5)	20.5 (14.3–26.4)	0.1115	621 (501–744)	656 (554–772)	0.3188	10.8 (9.2–12.3)	11.4 (8.1–14.3)	0.5902
CAD (N = 27)	13.7 (8.7–23.7)	20.6 (14.6–26.3)	0.0017	625 (538–733)	655 (551–772)	0.3635	11.0 (8.4–13.6)	11.3 (8.2–14.1)	0.6787
History of stroke (N = 11)	20.0 (12.8–31.8)	20.5 (14.1–26.1)	0.9470	745 (518–870)	625 (551–770)	0.2800	9.0 (7.6–12.3)	11.4 (8.2–14.2)	0.1102
Presence of cancer (N = 38)	16.7 (12.4–24.3	20.5 (14.4–26.2)	0.1387	727 (574–880)	650 (545–763)	0.0101	12.1 (9.6–14.1)	11.2 (8.1–14.1)	0.1743

CAD: coronary artery disease.

**Table 5:** Association between demographic, clinical, and biochemical parameters, and presenting at baseline one or less (N = 412) versus more than one (two to five) chronic disease (N = 88) in the studied population. Spearman’s rank correlation rho was calculated and presented in the univariate analysis. Stepwise logistic regression with a forward approach was used to discriminate variables independently associated with the presence of two or more diseases.

	Presence of >1 Diseases at Baseline				
	Univariate Analysis		Multivariate Logistic Regression Analysis		
	ρ	*p*	OR	95% CI	*p*
Age (years)	0.3213	0.0000	1.068	1.047–1.089	<0.001
Gender (f = 0/m = 1)	0.2403	0.0000	2.259	1.330–3.837	0.003
Living region North Italy (0) Central Italy (1) South Italy (2)	−0.1446	0.0012	-	-	-
Education (0 = primary, 1 = middle, 2 = high, or 3 = university)	−0.2378	0.0000	-	-	-
Job (0 = no, 1 = occasional, 2 = stable, 3 = retired, or 4= student)	0.1323	0.0030	-	-	-
BMI (Kg/m^2^)	0.2163	0.0000	1.069	1.010–1.131	0.021
Smoking (no = 0/y = 1)	−0.0297	0.5072			
Creatinine (mg/dL)	0.1967	0.0000	-	-	-
Uric acid (mg/dL)	0.2401	0.0000	-	-	-
Ca (mMol/L)	0.0232	0.6052			
P (mMol/L)	−0.1113	0.0127			
iPTH (pg/mL)	0.0654	0.1441			
WBCs (n * 1000/mL)	0.0126	0.7785			
Hemoglobin (g/dL)	0.0075	0.8666			
Platelets (n * 1000/mL)	−0.0633	0.1578			
25-OH-vitamin D (ng/mL)	−0.0795	0.0758	-	-	-
Vitamin A (mg/L)	0.1403	0.0017	1.003	1.001–1.131	<0.001
Vitamin E (mg/L)	0.0240	0.5919			

BMI: body mass index; WBCs: white blood cells; Ca: serum calcium, P: serum phosphorus; iPTH: serum parathormone.

**Table 6:** Association between demographic, clinical, and biochemical parameters (N = 500), with the presence of cancer at baseline in the studied population. Spearman’s rank correlation rho was calculated and presented in univariate analysis. Stepwise regression with a forward approach was used to discriminate variables independently associated with the presence of cancer.

	Presence of Cancer (N = 38)				
	Univariate Analysis		Multivariate Logistic Regression Analysis		
	ρ	*p*	OR	95% CI	*p*
Age (years)	0.1691	0.0001	1.044	1.019—1.069	<0.001
Gender (f = 0/m = 1)	0.1023	0.0222	-	-	-
Living region North Italy (0) Central Italy (1) South Italy (2)	−0.1057	0.0181	-	-	-
Education (0 = primary, 1 = middle, 2 = high, or 3 = university)	−0.0459	0.3054			
Job (0 = no, 1 = occasional, 2 = stable, 3 = retired, or 4= student)	0.0575	0.1991			
BMI (Kg/m^2^)	0.0649	0.1472			
Smoking (no = 0/y = 1)	−0.0907	0.0426	-	-	-
Creatinine (mg/dL)	0.0884	0.0482	-	-	-
Uric acid (mg/dL)	0.1017	0.0230	-	-	-
Ca (mMol/L)	−0.0270	0.5462			
P (mMol/L)	−0.0334	0.4556			
iPTH (pg/mL)	−0.0055	0.9030			
WBCs (n * 1000/mL)	−0.0221	0.6217			
Hemoglobin (g/dL)	−0.0185	0.6796			
Platelets (n * 1000/mL)	−0.0055	0.9021			
25-OH-vitamin D (ng/mL)	−0.0663	0.1389			
Vitamin A (mg/L)	0.1151	0.0100	1.002	1.000—1.004	0.014
Vitamin E (mg/L)	0.0608	0.1746			

BMI: body mass index; WBCs: white blood cells; Ca: serum calcium, P: serum phosphorus; iPTH: serum parathormone.

## Data Availability

The data presented in this study are available upon request from the corresponding author due to the privacy restrictions applied in our law.

## Supplementary Materials

The following supporting information can be downloaded at: https://www.mdpi.com/article/10.3390/nu18060943/s1.
