# Supplementary figures and images for: Serum Vitamin D, A, and E Concentrations and Their Associations with Chronic Diseases in Adult Patients Referred to Italian General Practitioners’ Offices
Source: Nutrients. 2026 Mar 17;18(6):943. doi: 10.3390/nu18060943 (PMC13029338; doi:10.3390/nu18060943)

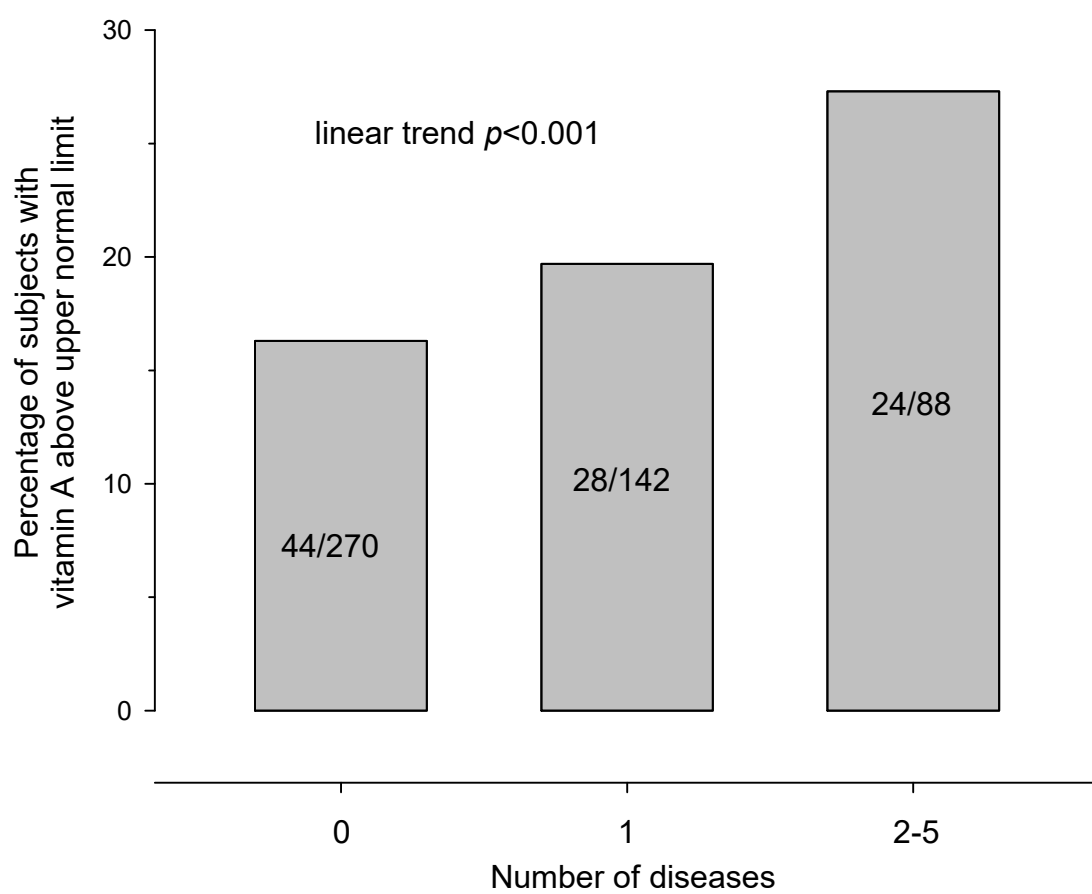

Supplement: Supplementary file 1 [file nutrients-18-00943-s001.zip › nutrients-4184556-supplementary.pdf]
